# IRF4 expression by lung dendritic cells drives acute but not Trm cell–dependent memory Th2 responses

**DOI:** 10.1172/jci.insight.140384

**Published:** 2022-11-08

**Authors:** Daniel F. Camacho, Tania E. Velez, Maile K. Hollinger, Esther Wang, Chanie L. Howard, Eli P. Darnell, Domenick E. Kennedy, Paulette A. Krishack, Cara L. Hrusch, Marcus R. Clark, James J. Moon, Anne I. Sperling

**Affiliations:** 1 Committee on Immunology and Department of Medicine and; 2 Pritzker School of Medicine, University of Chicago, Chicago, Illinois, USA.; 3 Department of Medicine, University of Virginia, Charlottesville, Virginia, USA.; 4 Massachusetts General Hospital, Boston, Massachusetts, USA.; 5 Harvard Medical School, Boston, Massachusetts, USA.

**Keywords:** Immunology, Asthma, Dendritic cells, Th2 response

## Abstract

Expression of the transcription factor interferon regulatory factor 4 (IRF4) is required for the development of lung conventional DCs type 2 (cDC2s) that elicit Th2 responses, yet how IRF4 functions in lung cDC2s throughout the acute and memory allergic response is not clear. Here, we used a mouse model that loses IRF4 expression after lung cDC2 development to demonstrate that mice with IRF4-deficient DCs display impaired memory responses to allergen. This defect in the memory response was a direct result of ineffective Th2 induction and impaired recruitment of activated effector T cells to the lung after sensitization. IRF4-deficient DCs demonstrated defects in their migration to the draining lymph node and in T cell priming. Finally, T cells primed by IRF4-competent DCs mediated potent memory responses independently of IRF4-expressing DCs, demonstrating that IRF4-expressing DCs are not necessary during the memory response. Thus, IRF4 controlled a program in mature DCs governing Th2 priming and effector responses, but IRF4-expressing DCs were dispensable during tissue-resident memory T cell–dependent memory responses.

## Introduction

Tissue-resident memory T cells (Trm cells) mediate inflammatory responses in various nonlymphoid tissues, and there are questions regarding the mechanisms underpinning the contribution of CD4^+^ Trm cells to allergic airway disease. Like circulating memory T cells, Trm cells develop in response to initial antigen exposure at mucosal surfaces and persist beyond the contraction phase of the immune response. However, Trm cells maintain proximity to barrier tissues where they are poised to respond rapidly upon subsequent antigen exposure (1). Trm cells can contribute to a “first-line” response until additional cells are recruited from the circulation and are often sufficient to control invading pathogens (2). However, how CD4^+^ Trm cells are activated within the lung milieu and the role of DCs in CD4^+^ Trm cell activation is not known. In settings in which T cell responses are pathogenic, such as in allergic asthma, these sentinel Trm cells may serve as central mediators of disease. Thus, investigating how Trm cells are generated, maintained, and restimulated is critical to understanding chronic allergic lung disease.

Many questions remain unanswered regarding how long-lived Trm cells are maintained and how they are reactivated during subsequent responses, particularly with regard to CD4^+^ Trm cells. While the pool of CD8^+^ Trm cells remains stable in the skin for up to 200 days after its establishment (3–6), lung CD8^+^ Trm cell numbers decline more rapidly and rely on replenishment from the circulating memory population or reintroduction of the inflammatory stimulus (6–9). Evidence regarding the duration of CD4^+^ Trm cell maintenance is limited, but one study showed that maintenance of vaginal CD4^+^ Trm cells against HSV2 was shorter than that of CD8^+^ Trm cells (10). Two studies have demonstrated declining allergen-specific CD4^+^ Trm cells in the lungs in the 70- to 90-day period following allergen challenge (11, 12). IL-2, IL-4, and IL-7 are implicated in the development and maintenance of lung CD4^+^ Trm cells in type 2 immunity during this time (11–13), but the source of these cytokines and whether these signals are sufficient remains to be elucidated.

Apart from requiring antigen presentation from a specific DC subset, it is possible that Trm cells are fully licensed to respond upon antigen presentation from any source or that Trm cells may be more reliant on innate or cytokine signals to prompt a response (14, 15). As in all mucosal tissues, numerous DC populations exist in the lungs. Specific transcription factors regulate DC development and function in acute immune responses. Conventional DCs (cDCs) in the lung are broadly divided into 2 groups: cDC1s, which express CD103, and cDC2s which express CD11b. The cells referred to as cDC1s, which depend on the transcription factors basic leucine zipper ATF-like transcription factor 3 (BATF3) and interferon regulatory factor 8 (IRF8), are proficient at cross-presentation as well as priming Th1 and specific Treg responses (16–19). Roles for cDC2s, which are dependent upon IRF4 for development, include promoting Th2, Th17, and specific Treg responses (20–29). The Th2-promoting IRF4-expressing cDC2 subset is also dependent on the transcription factor Kruppel-like factor 4 (KLF4) and expresses CD24, while the Th17-promoting cDC2 subset is dependent on Notch2 and lacks CD24 (ref. 24 and Supplemental Figure 1; supplemental material available online with this article; https://doi.org/10.1172/jci.insight.140384DS1). When mice lack IRF4 entirely or lack IRF4 during the CD11c^int^ pre-cDC stage, they fail to develop cDC2 populations in the lungs, lung-draining lymph nodes (LdLNs), small intestine, mesenteric lymph nodes, and spleen (25, 27, 30, 31). Because of the systemic failure of cDC2 development in the absence of IRF4, it has been challenging to study the ongoing role for IRF4 in mature DCs during inflammatory responses in vivo. To circumvent this, many studies have instead focused on the functional capacity of cultured bone marrow–derived DCs (BMDCs). To address this problem, we have developed a mouse model using a CD11c-Cre strain that does not express Cre until after the pre-DC stage, thereby only deleting IRF4 in mature DCs. Thus, these mice have normally developed cDC2s, but the mature cDC2s no longer express IRF4 (20). This mouse model has enabled us to isolate IRF4-dependent DC functions during immune responses in vivo.

Whether there is a role for tissue DCs in maintaining or reactivating CD4^+^ Trm cells in the lungs has yet to be determined. In a vaginal infection model, CD8^+^ Trm cells were still capable of proliferation in response to infection, despite depletion of CD11c^+^ cells, indicating that antiviral Trm cells can mediate a response in the absence of DCs (32). However, another vaginal infection model, in which CD8^+^ Trm cells were generated using the prime-and-pull technique, demonstrated that mice without MHC-I–expressing CD301b^+^ cDC2s in the vaginal lamina propria were much more susceptible to infection compared with mice that possess this DC subset (33). One study focusing on lung CD4^+^ Trm cells demonstrated that the proportion of lung cDC2s increases during an allergic memory response, with more DCs expressing CD86 (34). The activation of these DCs during memory inflammation suggests a possible unexplained role for cDC2s in the memory recall response. Thus, a more detailed understanding of how DCs support memory Trm cell responses is needed.

This study addresses fundamental questions regarding whether IRF4-expressing DCs regulate the development and recall response of type 2 Trm cells. Using our mice that have mature cDC2s, which lack IRF4 expression, we demonstrate that mice with IRF4-deficient DCs during sensitization exhibited diminished Trm cell–dependent memory responses to allergen. In addition, we demonstrate that IRF4 controlled IL-10 and IL-33 DC expression as well as migration to the draining lymph node during allergic sensitization. After finding that IRF4-expressing DCs were necessary during sensitization, we investigated the ongoing role for IRF4-expressing DCs in maintaining Trm cells that were primed by IRF4-competent DCs in WT hosts. T cells primed by IRF4-competent DCs were able to seed the lungs and mediate potent memory responses independently of IRF4-expressing DCs. In total, we found that IRF4 controls a program in mature DCs that governs Th2 priming during sensitization and Th2 effector responses during challenge and that impaired CD4^+^ Trm cell–dependent responses stem from earlier defects.

## Results

### IRF4-expressing DCs regulate the CD4^+^ Trm cell–restricted type 2 inflammatory memory response to HDM rechallenges.

**Figure 1:**
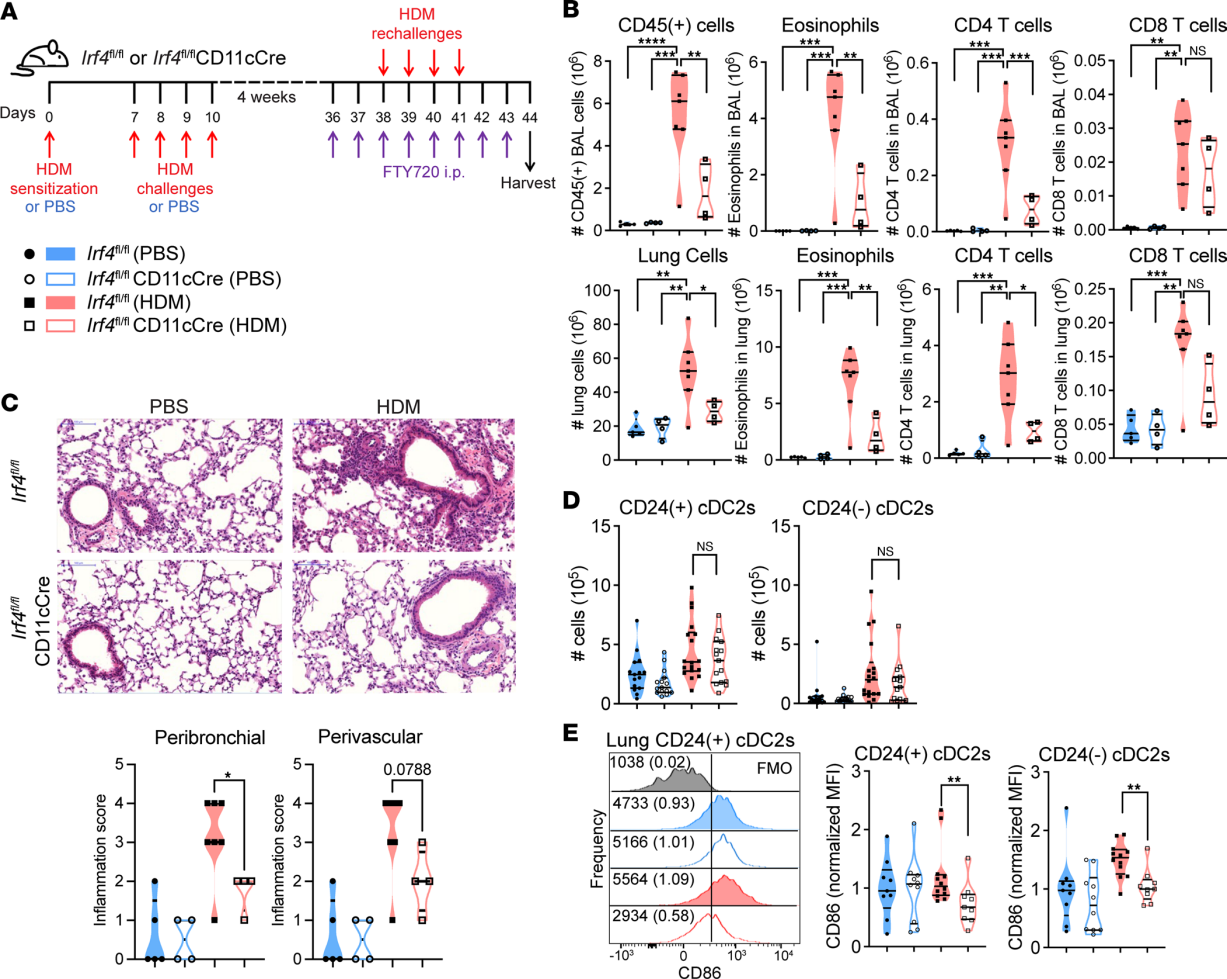
IRF4-expressing DCs regulate the Trm cell–restricted type 2 inflammatory memory response to HDM rechallenges. (**A**) Schematic of the experimental protocol for Trm cell–restricted memory response to HDM. (**B**) Total cellularity, eosinophils, CD4^+^ T cells, and CD8^+^ T cells in the airways (top) or lungs (bottom); *n* = 20. (**C**) H&E staining of the lungs confirms that mice with IRF4-deficient DCs are protected from allergic airway inflammation during the memory recall response to HDM; *n* = 20. Scale bar: 100 μm. (**D**) Number of CD24^+^ cDC2s and CD24^–^ cDC2s in the lungs; *n* = 63. (**E**) For lung CD24^+^ cDC2s and CD24^–^ cDC2s, MFI of CD86 normalized to the mean of the PBS-treated *Irf4^fl/fl^* group, with representative flow plots; *n* = 40. Data are (**B** and **C**) representative of or (**D** and **E**) combined from 3 independent experiments with *n* ≥ 4 mice per group in each experiment; statistics (**B**, ordinary 1-way ANOVA with Tukey’s multiple comparisons test; **C** and **D**, Mann-Whitney test) were performed in GraphPad Prism. Data are shown as the mean ± SEM (*P < 0.05; **P < 0.01; ***P < 0.001; ****P < 0.0001). Also see Supplemental Figures 2 and 4.

We previously reported that novel *Irf4^fl/fl^*CD11cCre mice, in which IRF4 is depleted after DC maturation, do not develop robust type 2 effector responses in the lungs following house dust mite (HDM) sensitization and challenge (20). However, it remains unknown whether these defects persist throughout subsequent memory recall responses or if the defective development of the type 2 response resolves over time. We sensitized and challenged *Irf4^fl/fl^ or Irf4^fl/fl^*CD11cCre mice with HDM and rechallenged the mice 4–5 weeks later. Rechallenges were performed while treating the mice with FTY720 to limit the recall response to lung Trm cells (Figure 1A and ref. 12), as others have shown that treatment with this sphingosine-1-phosphate receptor agonist downregulates S1P_1_ and thus retains circulating T cells in lymphoid organs (35). We confirmed that FTY720 treatment does not affect the lung Trm cell population but effectively depletes circulating T cells (Supplemental Figure 2, A–D). Mice lacking IRF4 in DCs exhibited a severely mitigated type 2 inflammatory Trm cell–dependent response to HDM rechallenge compared with their WT littermates, with fewer eosinophils and CD4^+^ T cells infiltrating the airways (as measured by cells in the bronchoalveolar lavage) or present in the lungs (Figure 1B). Indeed, evaluation of H&E-stained histological sections revealed that mice with IRF4-expressing DCs mounted robust inflammation in response to HDM compared with PBS, whereas the lungs of mice with IRF4-deficient DCs became less inflamed (Figure 1C).

An analysis of the lung DCs and their expression of costimulatory molecules revealed that IRF4-deficient CD24^+^ cDC2s and CD24^–^ cDC2s were present to an equal extent compared with their WT counterparts (Figure 1D), yet they expressed less CD86 (Figure 1E). None of the examined antigen-presenting cells (APCs) exhibited IRF4-dependent expression of CD80 (data not shown). This demonstrates that mice with IRF4-deficient DCs failed to mount memory type 2 responses and that their DCs were not appropriately activated during the recall response. Thus, *Irf4^fl/fl^*CD11cCre mice display diminished effector responses to HDM (20) and have defective memory type 2 responses.

### Lungs of mice with IRF4-deficient DCs contain fewer Der p 1–specific Th2rm cells during the memory phase.

**Figure 2:**
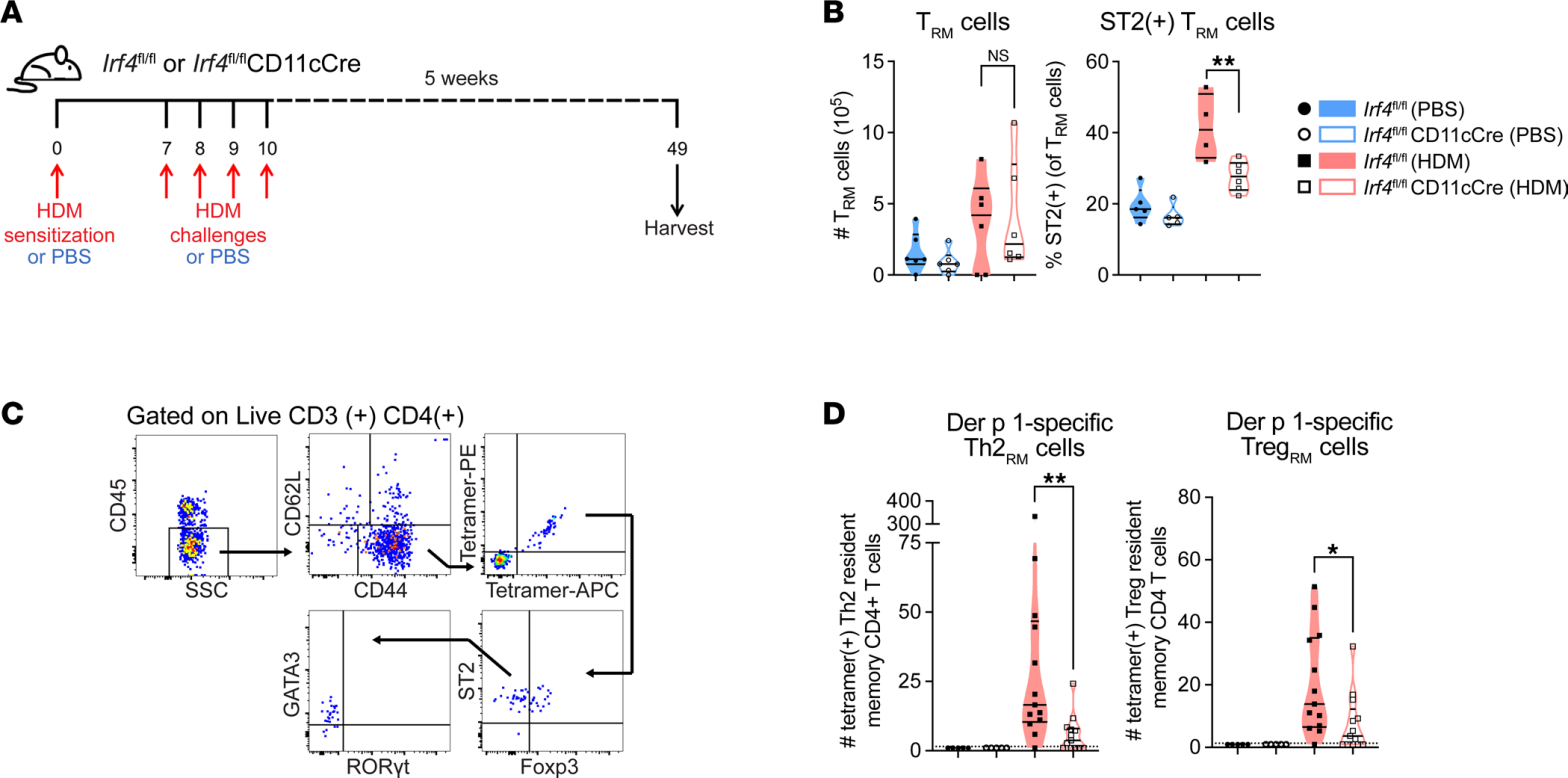
Lungs of mice with IRF4-deficient DCs contain fewer Der p 1–specific Th2rm cells during the memory phase. (**A**) Schematic of experimental protocol for resting memory lung analysis. (**B**) Number of lung Trm cells and the proportion expressing ST2; *n* = 20. Data are representative of 3 independent experiments with *n* ≥ 4 mice per group in each experiment. Analysis was performed by ordinary 1-way ANOVA with Tukey’s multiple comparisons test. (**C**) Gating of antigen-specific T cells and their expression of lineage-defining transcription factors. (**D**) Graphed flow plots show the number of tetramer^+^CD4^+^ Trm cells expressing GATA3 or Foxp3; *n* = 36. Three independent experiments (*n* ≥ 5 per group) were statistically significant. Two of these experiments combined are shown. Kruskal-Wallis test (with Dunn’s multiple comparisons test) was performed in GraphPad Prism. Data are shown as the mean ± SEM (*P < 0.05; **P < 0.01). Also see Supplemental Figures 3 and 4.

We hypothesized that the reduced memory response to HDM in *Irf4^fl/fl^*CD11cCre mice was due to a defect in the underlying CD4^+^ Trm cell pool that develops in response to sensitization and challenge. Without rechallenging the mice, we harvested the lungs 4–5 weeks after acute sensitization and challenge of *Irf4^fl/fl^* or *Irf4^fl/fl^*CD11cCre mice with HDM or PBS vehicle control (Figure 2A). To identify tissue-resident cells in the lung parenchyma, such as the Trm cells, we labeled the cells in circulation by intravenously injecting the mice with fluorescent anti-CD45 antibody minutes prior to sacrifice (36). CD4^+^ Trm cells were identified as CD3^+^CD4^+^CD44^hi^CD62L^lo^ cells that expressed CD69 and CD11a but did not display intravascular CD45 staining (Supplemental Figure 2, B–D). While no significant difference in the total number of CD4^+^ Trm, T central memory, or T effector memory (Tem), cells was evident in the absence of IRF4-expressing DCs (Figure 2B and Supplemental Figure 3, A–C), fewer CD4^+^ Trm cells in *Irf4^fl/fl^*CD11cCre mice expressed the IL-33 receptor (ST2), which is a marker of Th2 cells and a subset of Tregs (Figure 2B). We used tetramers to identify antigen-specific cells, using the tetramer on 2 fluorochromes to increase confidence that the cells specifically recognized the intended epitope, as opposed to the fluorochrome itself (Figure 2C and ref. 37). We found that *Irf4^fl/fl^*CD11cCre mice had fewer GATA3^+^ST2^+^ Der p 1–specific Trm cells and Foxp3^+^ Der p 1–specific Trm cells (Figure 2D), suggesting that decreased antigen-specific CD4^+^ Trm cells may limit memory Th2 and Treg responses.

**Figure 3:**
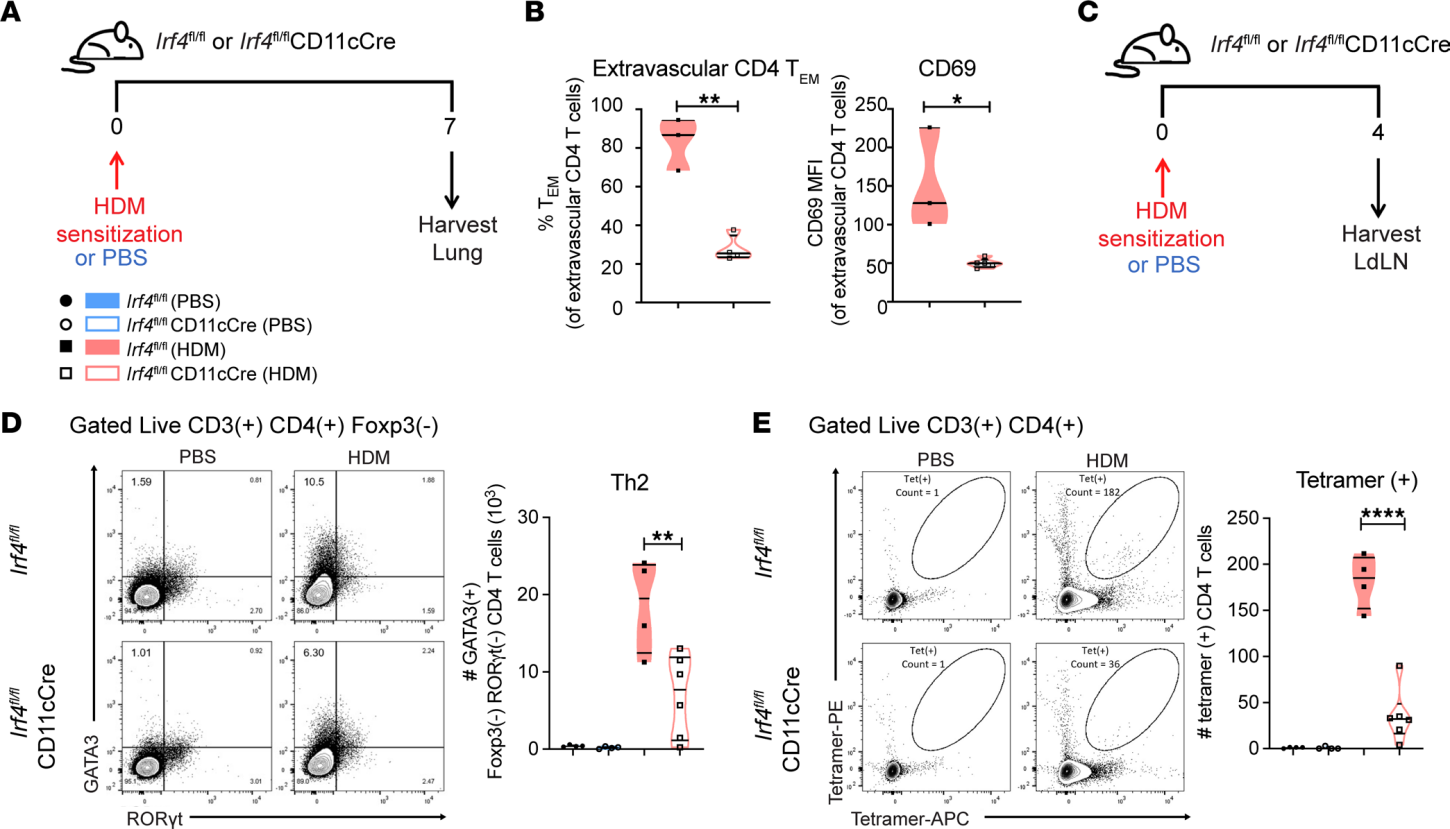
Mice with IRF4-deficient DCs are unable to adequately recruit Tem cells to the lungs and produce Th2 cells or tetramer^+^ T cells in the lung draining lymph nodes after HDM sensitization. (**A**) Schematic of experimental protocol for analysis of initiation of Th2 responses during sensitization phase in the lung. (**B**) Proportion of T effector cells of lung extravascular CD4^+^ T cells, and CD69 expression by extravascular lung T cells; *n* = 7. (**C**) Schematic of experimental protocol for analysis of initiation of Th2 responses during sensitization phase in the lung draining lymph nodes (LdLNs). (**D**) GATA3 and RORγt expression by LdLN T conventional cells on day 4 after HDM sensitization; *n* = 18. (**E**) Number of tetramer^+^ LdLN CD4^+^ T cells; *n* = 18. Data are representative of 2 independent experiments with *n* ≥ 3 mice per group; statistics (**B**, unpaired *t* test with Welch’s correction; **D** and **E**, ordinary 1-way ANOVA with Tukey’s multiple comparisons test) were performed in GraphPad Prism. Data are shown as the mean ± SEM (*P < 0.05; **P < 0.01; ****P < 0.0001).

To determine the stage at which Th2 defects begin, we sensitized *Irf4^fl/fl^ or Irf4^fl/fl^*CD11cCre mice to HDM and evaluated the T cells in the lung 7 days later (Figure 3A). This revealed a reduced proportion of lung parenchymal T effector cells and reduced expression of CD69 after HDM sensitization (Figure 3B), demonstrating a defect in the recruitment of activated T cells to the lungs after sensitization. We examined the expression of T cell lineage–specifying transcription factors in the LdLNs (Figure 3C) and found that the number of conventional Th2 cells was reduced in *Irf4^fl/fl^*CD11cCre mice by 4 days after sensitization (Figure 3D). Furthermore, the number of Der p 1–MHC-II tetramer^+^ CD4^+^ T cells was also reduced at this time point, demonstrating a diminished antigen-specific response during priming (Figure 3E). These data indicate that IRF4 is acting in DCs during the earliest stages of HDM sensitization to initiate type 2 lung responses.

### IRF4 regulates DC migration and priming of naive T cells during HDM sensitization.

**Figure 4:**
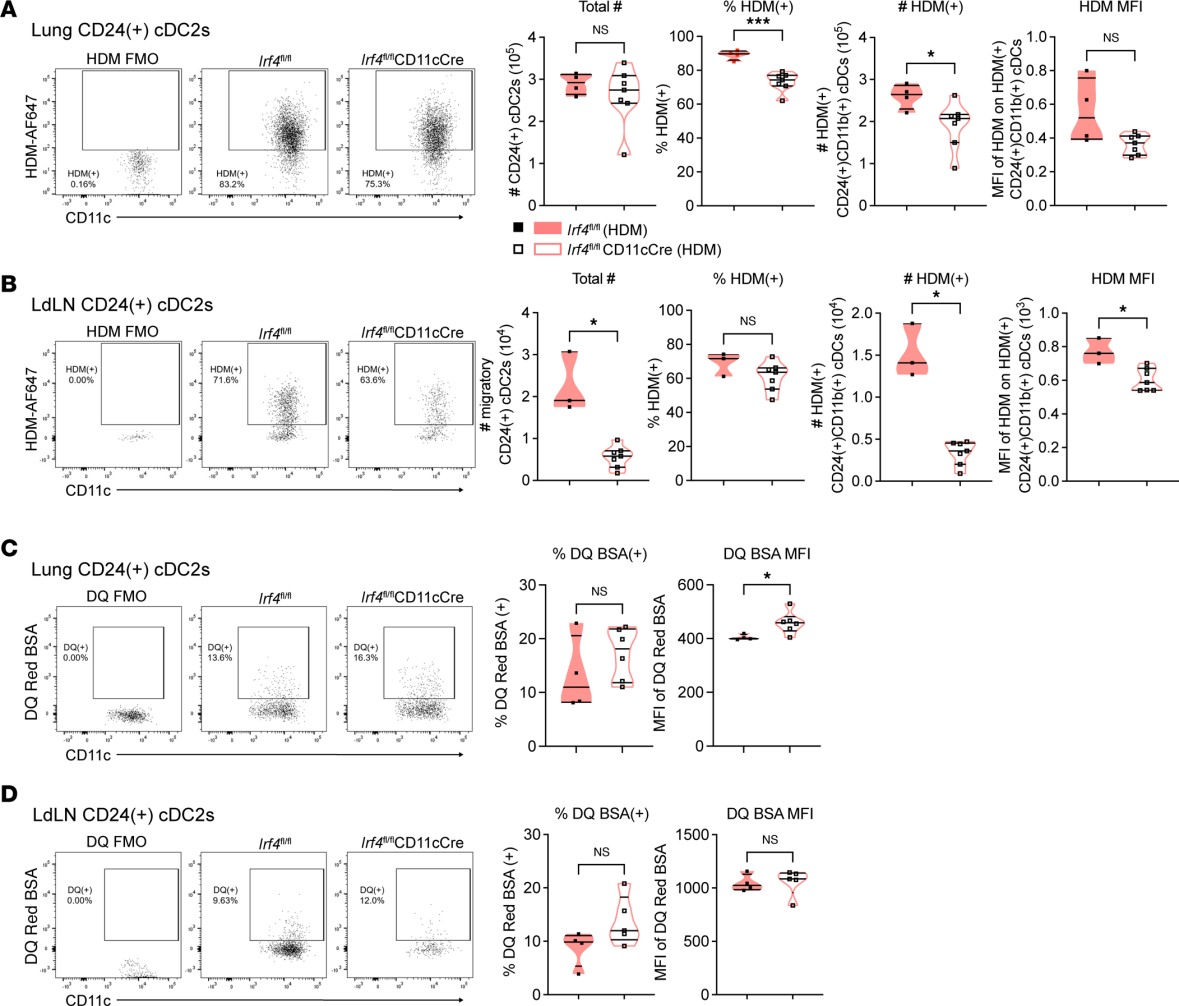
IRF4 regulates allergen phagocytosis and migration of CD24^+^ cDC2s to the lung draining lymph nodes during HDM sensitization. (**A** and **B**) Flow cytometry plots depict fluorescently labeled HDM in CD24^+^ cDC2s, quantified number of CD24^+^ cDC2s, the proportion and number that were HDM^+^, and MFI of HDM in either (**A**) the lungs (*n* = 11) or (**B**) lung draining lymph nodes (LdLNs; *n* = 10), as 1 LdLN could not be harvested. (**C** and **D**) Flow cytometry plots depict fluorescence of processed DQ Red BSA in CD24^+^ cDC2s, proportion DQ^+^, and MFI of DQ in either (**C**) the lungs (*n* = 10) or (**D**) LdLN (*n* = 9). Data are representative of 2 independent experiments with *n* ≥ 4 mice per group; statistics (unpaired *t* test with Welch’s correction) were performed in GraphPad Prism. Data are shown as the mean ± SEM (*P < 0.05; ***P < 0.001). Also see Supplemental Figure 5.

We hypothesized that IRF4 regulates particular DC processes required to initiate Th2 responses during sensitization in vivo. A first step in initiating the immune response is the phagocytosis of allergens by DCs. To assess allergen phagocytosis, we sensitized mice with fluorescently labeled HDM and evaluated lung DCs the next day. There was a slight but statistically significant reduction in the proportion and number of HDM-bearing CD24^+^ cDC2s in the lungs of *Irf4^fl/fl^*CD11cCre mice compared with that in *Irf4^fl/fl^* mice (Figure 4A). There was no difference in the overall number of any DC subset (Figure 4A and Supplemental Figure 4A). The CD24^–^ cDC2 subset, which is also IRF4 dependent, had a slight but statistically significant reduction in the proportion that were HDM^+^ (Supplemental Figure 4B) but no reduction in cell number (Supplemental Figure 4C) or HDM MFI (Supplemental Figure 4D). Thus, while there is a small difference in allergen uptake in the IRF4-deficient lung cDC2s, allergen uptake is largely independent of IRF4.

It has been previously described that IRF4 is needed for DC expression of CCR7 and subsequent migration to the tissue-draining lymph nodes in the skin (30, 31). Thus, we found an expected reduction in the number of migratory CD24^+^ cDC2s in the LdLNs of *Irf4^fl/fl^*CD11cCre mice (Figure 4B). While the proportion of CD24^+^ cDC2s that were HDM^+^ was equal in the LdLNs between *Irf4^fl/fl^* and *Irf4^fl/fl^*CD11cCre mice, there was a reduction in the number of HDM^+^ DCs in the *Irf4^fl/fl^*CD11cCre mice (Figure 4B). No consistent defects were seen for other APC subsets lacking IRF4 in the LdLNs (Supplemental Figure 4, E–H). Thus, as was previously described for skin DCs (30, 31), the migration of IRF4-deficient lung CD24^+^ cDC2s to the lymph node is impaired. However, some allergen-bearing DCs are nevertheless capable of reaching the lymph nodes.

**Figure 5:**
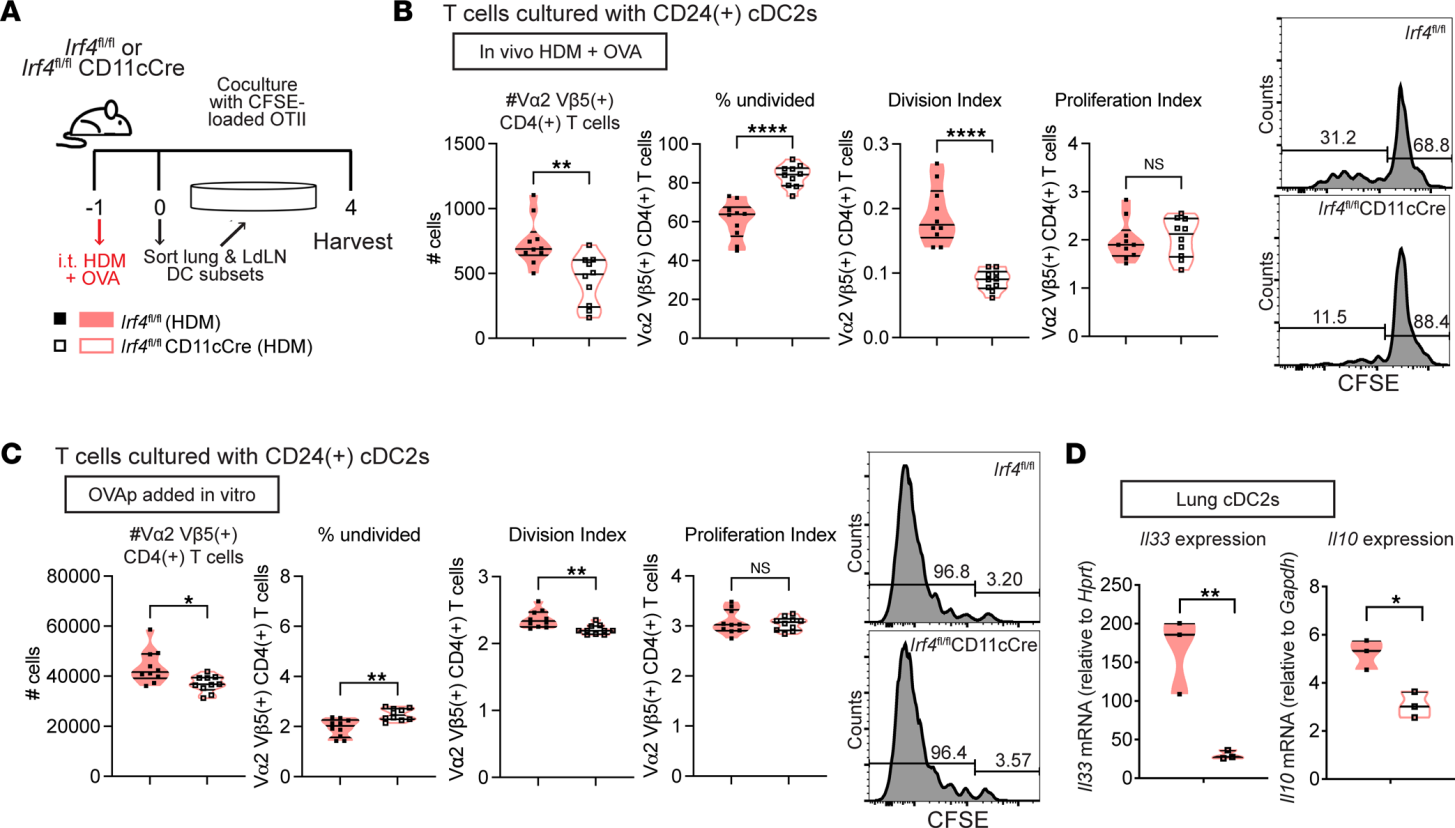
Ex vivo–sorted CD24^+^ cDC2s require IRF4 for robust T cell priming in vitro. (**A**) Schematic of experimental protocol for in vivo sensitization to HDM^+^ OVA, DC sorting, and in vitro coculture with CFSE-labeled T cells from naive OTII mice. (**B** and **C**) Number of OTII cells after culture, percentage undivided, division index, proliferation index, and CFSE dilution histograms for (**B**) in vivo HDM^+^OVA–sensitized CD24^+^ cDC2s (*n* = 20) or (**C**) those with OVA_323–339_ peptide added (*n* = 20). (**B** and **C**) Data are representative of 2 independent experiments with *n* ≥ 4 wells per group; statistics (unpaired *t* test with Welch’s correction) were performed in GraphPad Prism. (**D**) IL-33 and IL-10 expression by qPCR of sorted lung cDC2s after in vivo HDM sensitization; *n* = 6. Data represent 1 experiment with *n* = 3 mice per group; statistics (unpaired *t* test) were performed in GraphPad Prism. Data are shown as the mean ± SEM (*P < 0.05; **P < 0.01; ****P < 0.0001). Also see Supplemental Figure 6.

These findings raised the question of whether reduced Th2 responses in *Irf4^fl/fl^*CD11cCre mice are solely due to a reduced quantity of DCs reaching the lymph nodes. Using BMDCs, we previously showed that IRF4-deficient DCs display a reduced capacity to promote Th2 differentiation in vitro (20). Thus, we hypothesized that IRF4 regulates DCs processes beyond migration. One such function is the ability to process antigens upon phagocytosis. To assess this capability, we sensitized mice to HDM mixed with the surrogate reagent DQ Red BSA, which becomes fluorescent upon proteolytic cleavage. IRF4-deficient CD24^+^ cDC2s were capable of processing antigens both in the lungs (Figure 4C) and in the LdLNs (Figure 4D). To address whether there is a cell-intrinsic defect in the ability of IRF4-deficient DCs to prime T cells, we turned to ex vivo cultures where the number of DCs can be normalized. After HDM plus OVA sensitization, pooled lung and LdLN DCs from *Irf4^fl/fl^* or *Irf4^fl/fl^*CD11cCre mice were sorted and used to stimulate CFSE-labeled T cells from OTII T cell receptor–transgenic mice (Figure 5A). We found that ex vivo IRF4-deficient CD24^+^ cDC2s were less effective at inducing T cell proliferation (Figure 5B). Even when OVA peptide was added to the culture, the IRF4-deficient DCs were still slightly deficient in stimulating OTII proliferation (Figure 5C). Cultures with IRF4-deficient DCs produced fewer OTII cells with a greater proportion of undivided cells leading to a reduced division index. The proliferation index, which indicates the number of divisions undergone by cells that have entered cell division, was unchanged, suggesting that IRF4-expressing DCs are important for prompting T cell division but that once T cells divide, they do so to an equal extent. While CD24^–^ cDC2s had similar trends in these measures, their ability to induce cell division was inferior to that of CD24^+^ cDC2s (*P* = 0.0002), suggesting that CD24^–^ cDC2s are not well-suited to T cell priming in response to HDM (Supplemental Figure 5).

That IRF4-deficient CD24^+^ cDC2s are intrinsically less capable of priming T cells suggests that there are downstream effectors of IRF4 in DCs responsible for driving allergic T cell responses. Our previous in vitro work demonstrated that IRF4-deficient DCs express reduced IL-33 and IL-10 (20). To determine whether IRF4 regulates IL-33 and IL-10 expression in vivo, we sensitized *Irf4^fl/fl^* and *Irf4^fl/fl^*CD11cCre mice to HDM, sorted lung cDC2s 18 hours later, and evaluated IL-33 and IL-10 expression by qPCR. *Il33* and *Il10* expression by lung cDC2s, as assessed by qPCR, was dependent on IRF4 during in vivo HDM sensitization (Figure 5D). This suggests that IRF4 controls a pro-Th2 program involving these factors, culminating in Th2 polarization.

### IRF4 expression in DCs is not required for CD4^+^ Trm cell maintenance or recall responses.

**Figure 6:**
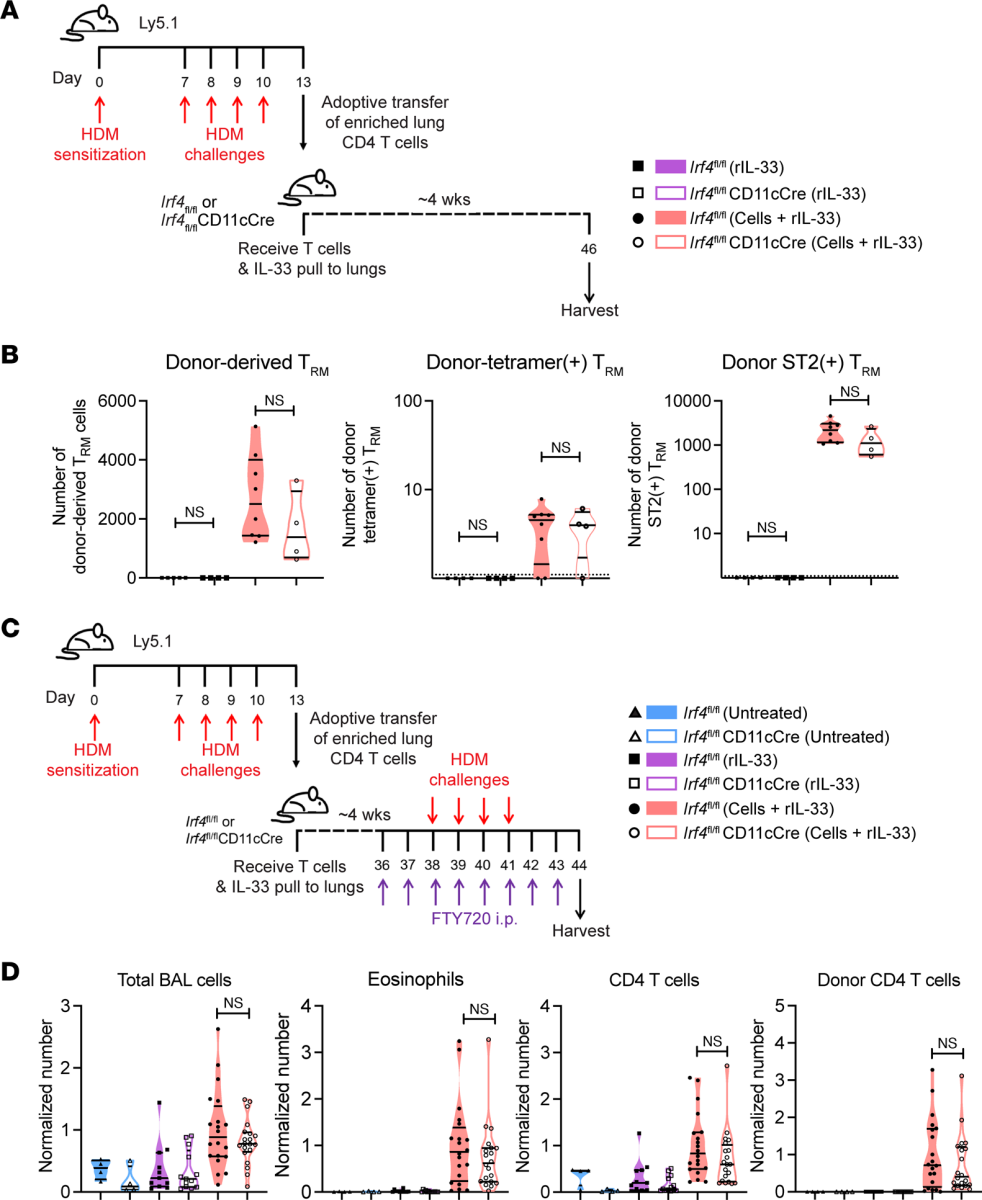
IRF4 expression in DCs is not required for CD4^+^ Trm cell maintenance or recall responses. (**A**) Ly5.1 mice were sensitized and challenged with HDM. Lung Tem cells were adoptively transferred to *Irf4^fl/fl^* or *Irf4^fl/fl^*CD11cCre mice and pulled to the lungs with intratracheal rIL-33 in the “Cells + rIL-33” group. Control groups received no cells and either rIL-33 alone or no treatment at all. Lungs were harvested after 4 weeks. (**B**) Quantified number of donor-derived Trm cells, donor-derived tetramer^+^ Trm cells, and ST2^+^ donor-derived Trm cells. Data represent 1 experiment with *n* ≥ 4 mice per group and a total of *n* = 21; statistics (Mann-Whitney test) were performed in GraphPad Prism. Data are shown as the mean ± SEM. (**C**) As in **A**, but followed by HDM challenges during FTY720 treatment. (**D**) Number of total cells, eosinophils, CD4^+^ T cells, and donor CD4^+^ T cells in the airways normalized to the mean of the *Irf4^fl/fl^* group receiving donor cells. Data represent 4 combined experiments with *n* ≥ 4 mice per group and a total of *n* = 52; statistics (Mann-Whitney test) were performed in GraphPad Prism. Data are shown as the mean ± SEM. Also see Supplemental Figure 6.

Having demonstrated early defects in the immune response when DCs lack IRF4, the question remained of whether the impaired memory response observed was solely attributable to these early defects or whether IRF4-expressing DCs played an ongoing role in sustaining the allergic response. We circumvented early defects in T cell priming and differentiation by sensitizing and challenging WT CD45.1 mice with HDM, isolating CD4^+^ T cells from the inflamed lungs, and then adoptively transferring these lung T cells into either *Irf4^fl/fl^* or *Irf4^fl/fl^*CD11cCre mice (Figure 6A). Recruitment of the transferred cells was assisted by “pulling” with intratracheal instillation of rIL-33, which induces lung expression of chemokines (38). The mice were then rested for 4 to 5 weeks to allow for contraction of the adoptively transferred effector CD4^+^ T cells into Trm cells. Polyclonal and tetramer^+^ donor Trm cells were present to an equal extent in the lungs of both *Irf4^fl/fl^* and *Irf4^fl/fl^*CD11cCre mice (Figure 6B), demonstrating that T cells primed by IRF4-expressing DCs were capable of taking up residence and persisting for many weeks without the continued presence of IRF4-expressing DCs. We found a modest reduction in allergen uptake and CD86 expression by CD24^+^ cDC2s in this adoptive transfer model (Supplemental Figure 6, A–C), similar to that in the aforementioned experiments, validating our use of this T cell adoptive transfer to investigate IRF4-dependent cDC2 defects.

To determine the role of IRF4-expressing DCs during recall responses, we rechallenged the mice with HDM during simultaneous FTY720 treatment to restrict the memory response to the lung Trm cells (Figure 6C). Upon challenge, *Irf4^fl/fl^*CD11cCre mice displayed allergic responses equal in magnitude to those mounted by *Irf4^fl/fl^* littermates, as they were equally capable of recruiting eosinophils and CD4^+^ T cells to the airways (Figure 6D). There were equal numbers of the donor CD4^+^ T cells in the bronchoalveolar lavage, suggesting that their memory response was similar whether the lung DCs were IRF4 deficient or IRF4 sufficient. Taken together, our data demonstrate that IRF4-expressing DCs are necessary for the development of the effector Th2 response but not for reactivating resting Trm cells.

## Discussion

In this study, we found that mature IRF4-expressing CD24^+^ cDC2s play numerous crucial roles in the priming and differentiation of naive T cells in response to HDM in vivo. IRF4-deficient CD24^+^ cDC2s display minor defects in their ability to phagocytose inhaled allergens, no defect in antigen processing, and reduced capacity for migration to lymph nodes. Beyond this quantitative deficiency, IRF4-deficient CD24^+^ cDC2s display defects in their capacity to prime naive T cells and deliver additional signals such as IL-10 and IL-33 during sensitization. Intriguingly, we found that mature DCs need not express IRF4 during the homing of Th2 Tem cells to the lungs, the development of Th2 Tem cells into Th2 Trm cells (Th2rm cells), or the subsequent persistence of Th2rm cells in the lungs. Finally, Th2rm cells that have been educated by IRF4-expressing DCs are able to orchestrate the infiltration of eosinophils and CD4^+^ T cells to the airways, despite the absence of IRF4-expressing CD24^+^ cDC2s during the recall response.

Our study pinpoints numerous IRF4-dependent functions for mature lung DCs in educating Th2 cells during in vivo sensitization. We were able to study these effects in vivo, because, in contrast to previous mouse models where deletion of IRF4 globally or in the entire CD11c^+^ compartment causes the absence of lung, intestine, lymph node, and spleen CD24^+^ cDC2s (25, 27, 30, 31,39), we found that IRF4-deficient CD24^+^ cDC2s were present to a normal extent in the lungs and spleens of our mice (Figure 4A and ref. 20). The CD11c-Cre mice used in this study excise IRF4 only after the pre-cDC stage, thereby producing mature IRF4-deficient cDC2 cells (20). While other models have not been useful for studying IRF4-deficient lung DCs in vivo, dermal DCs could still develop independently of IRF4. In fact, one study found increased numbers of dermal CD11b^+^ DCs due to IRF4-dependent deficiency in CCR7 and reduced drainage to the skin-draining lymph nodes (30). In our mouse model, lymph node homing of migratory CD24^+^ cDC2s was severely limited in the context of IRF4 deficiency (Figure 4B). However, this did not appear to cause an increase in the number of these DCs in the lungs (Figure 4A).

IRF4-deficient BMDCs have inferior T cell–priming capabilities due to defects in antigen processing and presentation pathway genes, such as cathepsin S (*Ctss*), *H2-Oa*, *H2-DMb2*, *Ciita*, and *Cst3* (25). In our study, IRF4-deficient lung DCs could process exogenous antigen and even had higher levels of antigen processing as measured by BSA-DQ fluorescence (Figure 4C). Another study examining splenic cDC1s deficient in BATF3, a lineage-defining transcription factor for cDC1, similarly found that this genetic deletion led to increased OVA-DQ processing (40). This may suggest that the antigen-processing pathway is dysregulated when cDC2s lack IRF4 during development and that cDC2s capable of expressing IRF4 during development process antigens independently of IRF4 once mature. Alternatively, this may highlight a difference between in vitro–generated DC cultures and in vivo tissue-derived DCs.

Our findings suggest particular roles for IL-10 and IL-33 in Th2 differentiation. A recent report has corroborated the IRF4-dependent production of IL-10 by lung DCs (41). While other cells may produce these factors in response to a type 2 inflammatory stimulus, DCs are uniquely capable of migrating to particular microanatomic areas of the tissue-draining lymph nodes (42). Their ability to position themselves at the T cell–B cell border, a site for the education of Th2 cells, suggests that they are particularly unique messengers for delivering these signals (43). This may be especially important for short-range delivery of the cytokine IL-33, which is otherwise entirely bound by the soluble decoy receptor sST2 (44, 45) or is inactivated by oxidation (46). Previous studies have indicated that IRF4-expressing DCs play a role in the early education of Th2 cells, Tregs, and CD8^+^ Trm cells (20, 25, 26, 41). HDM sensitization and challenge generate a pool of CD4^+^ Trm cells but less potently induce CD8^+^ Trm cells (34). We found that HDM sensitization promotes allergen-specific CD4^+^ Trm cells with both Th2 and Treg subsets and that the absence of IRF4-expressing DCs results in a more significant loss of the Th2 subset. This loss of Th2 cells may result from diminished downstream mediators of IRF4 such as IL-33 and IL-10.

We have demonstrated that IRF4 expression by DCs is not necessary for the maintenance and recall response of Th2 cells that IRF4-expressing DCs previously primed. In particular, adoptively transferred Tem cells from mice sensitized and challenged to HDM were capable of homing to the lungs and persisting as Trm cells in the absence of allergen or IRF4-expressing DCs. Upon reintroduction of allergen, these Trm cells were able to mediate a type 2 response without assistance from circulating cells or IRF4-expressing DCs. It was previously shown that antigen-specific CD8^+^ Trm cells could proliferate in response to an LCMV peptide in the female reproductive tract when CD11c^+^ cells were depleted (32). MHC-II–expressing epithelial cells have also been shown to present antigen to lung CD4^+^ Trm cells (47). Together with our data that IRF4-expressing DCs are expendable for recall responses, these studies suggest that other nonclassical APCs can present antigen and stimulate appropriately educated Trm cells. Alternatively, Trm cells could be licensed to conduct an allergic recall response by the presence of allergen-triggered cytokines. The latter possibility is consistent with previous investigations pointing to tissue-derived signals, such as thymic stromal lymphopoietin (TSLP), IL-25, and IL-33, as important for licensing lung Th2 cells (48), particularly the requirement and sufficiency of IL-33 signaling in memory Th2 cells for IL-5–mediated eosinophilic responses (48). Indeed, our data indicate that the Der p 1 tetramer-specific T cells display nearly uniform expression of the IL-33 receptor ST2. Although we tracked the T cell response to the immunodominant epitope of HDM, there remain unexamined tetramer^–^ cells with other HDM-relevant specificities, which may follow similar patterns. For instance, nearly all microbe-specific clonal effector populations give rise to their own pool of long-lived memory cells (49). As such, it would be of interest to evaluate whether the ST2^+^ T cell compartment harbors the allergen-specific T cells of specificities other than for Der p 1 and whether these cells conduct an allergic response to IL-33 alone.

Together, these findings demonstrate that IRF4 controls a program in mature CD24^+^ cDC2s that governs Th2 priming during sensitization with profound implications for Th2 effector responses during challenge and that impaired Trm cell–dependent memory responses when DCs lack IRF4 stem from defects in earlier T cell education.

## Methods

### Mice.

C57BL/6 mice (WT) were purchased from Harlan Laboratories. B6 CD45.1 (B6.SJL-*Ptprc^a^Pepc^b^*/BoyJ, stock 002014; refs. 50–52) and conditional *Irf4* mutant (*Irf4^fl/fl^*; B6.129S1-*Irf4^tm1Rdf^*/J, stock 009380; ref. 53) mice were purchased from The Jackson Laboratory. *Irf4^fl/fl^* mice were bred to CD11c-Cre-GFP–transgenic mice [CD11cCre; C57BL/6J-Tg(*Itgax-cre,-EGFP*)4097Ach/J, stock 007567; ref. 54], which were developed and provided by Alexander Chervonsky (University of Chicago). OTII mice were bred and maintained at the University of Chicago. In all experiments, mice were matched for sex and age and blinded by ear tagging. Animals were bred and housed in a specific pathogen–free facility maintained by the University of Chicago Animal Resources Center.

### HDM-induced mouse model of allergic airway disease.

HDM extract (Stallergenes Greer, XPB82D3A25) was resuspended in sterile PBS. In sensitization-only experiments, mice were administered 100 μg HDM via intratracheal instillation on day 0 and were sacrificed 12–18 hours later. Lungs and/or draining lymph nodes were made into single-cell suspensions by mechanical disruption followed by digestion in 600 U/mL collagenase IV (MilliporeSigma, C5138) and 20 μg/mL DNase I (Worthington DP grade) for 1 hour at 37°C and then additional mechanical disruption and red blood cell lysis. In experiments requiring fluorescent antigen tracking, HDM was labeled with Alexa Fluor 647 Protein Labeling Kit (Life Technologies, A20173) per the manufacturer’s instructions. To measure antigen-processing capacity of lung DCs, mice were administered HDM mixed with the surrogate reagent DQ Red BSA (Thermo Fisher Scientific). In experiments requiring allergen challenge, mice were administered 25 μg HDM via intratracheal instillation on days 7, 8, 9, and 10 and then sacrificed on day 13. In resting memory experiments, resting mice were sacrificed after 4–5 weeks. At the time of sacrifice, the mice received an intravenous injection of biotinylated or PE-conjugated anti-CD45 (clone 30-F11, BioLegend). After 5 minutes, the lungs were perfused and harvested to allow for identification of CD45iv^+^ cells located in the vasculature or CD45iv^–^ cells in the lung parenchyma. In memory rechallenge experiments, mice were rechallenged during treatment with FTY720 (Enzo Life Sciences, BML-SL-140). Mice were pretreated daily for 2 days with 25 μg FTY720, delivered by intraperitoneal injection. Mice continued FTY720 treatment while receiving 25 μg HDM via intratracheal instillation daily for 4 days. The mice continued FTY720 treatment until sacrifice 3 days later. Analysis of cells in the airways was conducted by bronchoalveolar lavage, in which sterile PBS was used to wash the airways 4 times via a tracheal cannula for a total recovery of approximately 3.0 mL. For histologic evaluation of lung inflammation, the left lobe was fixed in 10% neutral buffered formalin and then paraffin-embedded, cut into 5 μm sections, and stained with H&E by the University of Chicago Human Tissue Resource Center. When indicated, recombinant mouse IL-33 (BioLegend, 580506) was used in murine experiments.

### Tetramer production.

As previously described, I-A^b^ containing the Der p 1 peptide 117–127 (CQIYPPNVNKI) was biotinylated and tetramerized with streptavidin-PE or streptavidin-APC (Prozyme) (55, 56). Tetramers were produced at Massachusetts General Hospital and shipped to the University of Chicago.

### T cell enrichment for adoptive transfer.

CD45.1 mice were sensitized and challenged as described above. On day 13, single-cell suspensions of lung cells were prepared as described above. Hematopoietic cells were isolated at the interface of 44% and 67% Percoll PLUS solutions (GE Healthcare, 17-5445-01). CD4^+^ T cells were then enriched using the manufacturer’s instructions with a MACS mouse CD4^+^ T cell isolation kit (Miltenyi Biotec, 130-104-454) to yield a purity of 90%–95% CD4^+^ T cells, which were washed and resuspended in PBS for intravenous adoptive transfer of approximately 1 × 10^6^ cells per mouse. Recipient mice were promptly intratracheally instilled with 100 ng recombinant mouse IL-33.

### T cell enrichment, labeling, and coculture.

Total lymph node and spleen cells were isolated from OTII mice and passed through a nylon wool column. Cells were then labeled with CFSE and cocultured with sorted lung DCs at a DC/T cell ratio of 1:10 for 4 days in 96-well round-bottomed plates.

### Flow cytometric analysis.

For staining of DCs, 1 × 10^6^ cells were suspended in 200 μL of staining buffer (PBS with 0.1% sodium azide and 2% BSA) and incubated for 5 minutes with 20 μL 2.4G2 supernatant. For staining of other cells, 5 × 10^5^ cells were suspended in 100 μL of staining buffer and incubated for 5 minutes with 10 μL 2.4G2 supernatant. Antibodies used include those in Supplemental Table 1.

For intracellular cytokine staining, cells were incubated in culture medium with 10 ng/mL phorbol 12-myristate 13-acetate and 500 ng/mL ionomycin for fixing with 2% formaldehyde overnight at 4°C. They were then permeabilized with 0.5% saponin in PBS containing 0.1% sodium azide and 2% BSA and then incubated with the indicated antibodies. For intracellular transcription factor staining, samples were prepared using the eBioscience Foxp3/Transcription Factor Staining Buffer Set (eBioscience, 00-5523-00) following the manufacturer’s instructions. Flow cytometric analysis was conducted using an LSR Fortessa (BD Biosciences) and Aurora (Cytek). The data were analyzed using FlowJo software (Tree Star Inc.). Cell sorting was conducted using a FACSAria III (BD Biosciences). These instruments are maintained by the Flow Cytometry and Antibody Technology Core Facility at the University of Chicago.

Lung DCs were gated according to the following strategy (Supplemental Figure 1). After gating out of SiglecF^+^ eosinophils and alveolar macrophages, lung DCs were defined as CD11c^+^ and MHC-II^hi^. This also excluded lung interstitial macrophages, which are CD11c^–^ (57). The DCs in the lung either expressed CD103 or CD11b, except for a small population of pDCs. The CD103^+^ DCs are cDCs known as cDC1. CD11b^+^ DCs include populations of CD88^+^ monocyte-derived DCs (58), which have also previously been defined by Ly6C, CD64 (FcγRI), and MAR-1 (FcεR1α) (22). The CD11b^+^CD88^–^ cDCs are all thought to be dependent on IRF4 but remain heterogeneous and can be further subdivided by CD24 expression. The CD24^+^ subset is known to be KLF4 dependent and has been suggested to be the primary subset responsible for type 2 responses (24). We conducted subsequent analyses for each of the following DC populations: monocyte-derived DCs, CD103^+^ cDCs, CD24^+^ cDC2s, and CD24^–^ cDC2s.

### qPCR.

RNA was isolated from sorted cells using a Quick-RNA Microprep Kit (Zymo Research, R1050), and cDNA was created for each sample using the High Capacity cDNA Reverse Transcription Kit (Thermo Fisher Scientific, 4368814), according to the manufacturer’s instructions. qPCR was conducted on a Bio-Rad CFX96 qPCR detection system, with conditions including denaturation at 95°C for 2 minutes and then 30 cycles of 95°C for 30 seconds, 53°C for 30 seconds, and 72°C for 40 seconds. C_T_ values were normalized to the housekeeping genes *Hprt* or *Gapdh*. PCR primers were as indicated: *Gapdh*, forward 5′-TTCACCACCATGGAGAAGGC-3′, reverse 5′-GGCATGGACTGTGGTCATGA-3′; *Hprt*, forward 5′-TGATCAGTCAACGGGGGACA-3′, reverse 5′-TTCGAGAGGTCCTTTTCACCA-3′; *Il10*, forward 5′-GCCAAGCCTTATCGGAAATGATCC-3′, reverse 5′-CACAGGGGAGAAATCGATGACAG-3′; and *Il33*, forward 5′-CTGCGTCTGTTGACACATT-3′, reverse 5′-CACCTGGTCTTGCTCTTGGT-3′.

### Statistics.

GraphPad Prism software was used for statistical analyses, and *P* values of less than 0.05 were considered significant. When data points came from a normal distribution, an unpaired Student’s 2-tailed *t* test was used to analyze experiments with 2 groups, and an ANOVA with Tukey’s or Holm-Sidak’s post test was used for the comparison of more than 2 groups. A 1-way ANOVA was used when there was 1 independent variable and 2-way ANOVA was used when there was more than 1 independent variable. Otherwise, a Mann-Whitney *U* test was performed for the comparison of 2 groups or a Kruskal-Wallis test with Dunn’s multiple comparisons test was performed for comparison of more than 2 groups. Bars represent the mean, and error bars represent the SEM.

### Study approval.

The University of Chicago Animal Resources Center approved all animal procedures. Studies conformed to the principles set forth by the Animal Welfare Act and the NIH guidelines for the care and use of animals in biomedical research.

## Author contributions

DFC and AIS designed the research studies. DFC, TEV, MKH, EW, and CL Howard, EPD, PAK, and CL Hrusch conducted experiments and acquired data. DFC, TEV, MKH, EW, CL Howard, DEK, and EPD analyzed data. JJM provided reagents. DFC, TEV, MKH, MRC, and AIS wrote the manuscript.

## Supplementary Material

Supplemental data

## Version 1 10/04/2022

In-Press Preview

## Version 2 11/08/2022

Electronic publication
