# Supplementary material for: IRF4 expression by lung dendritic cells drives acute but not Trm cell–dependent memory Th2 responses
Source: JCI Insight. 2022 Nov 8;7(21):e140384. doi: 10.1172/jci.insight.140384 (PMC9675458; doi:10.1172/jci.insight.140384)
Supplement: Supplemental data [file jciinsight-7-140384-s119.pdf]

Figure S1

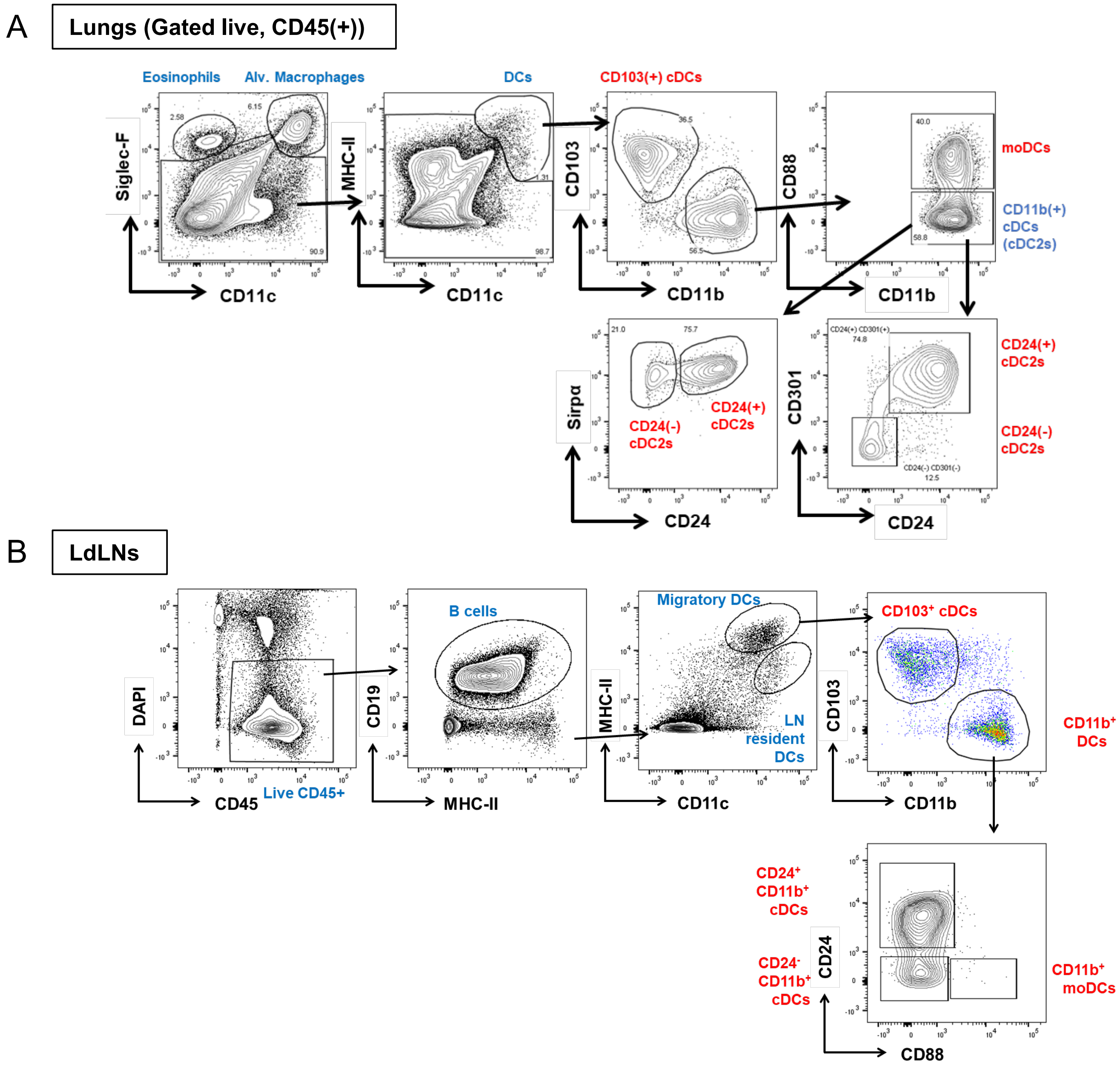

Figure S1, related to Materials & Methods and Introduction: Lung (A) and lung-draining lymph node (B) DC gating. After exclusion of Siglec-F(+) eosinophils and alveolar macrophages, lung DCs are defined as CD11c(+) and MHC-II high. This excludes interstitial macrophages, which are CD11c(-). DCs in the lung either express CD103 or CD11b, apart from a small population of pDCs expressing neither. The CD103(+) population are cDC1s. The CD11b(+) DCs include populations of CD88(+) monocyte-derived DCs, CD24(-) conventional DC2s, and CD24(+) conventional DC2s. As MoDC are not known to be migratory, very few are found in the LdLN.

Figure S2

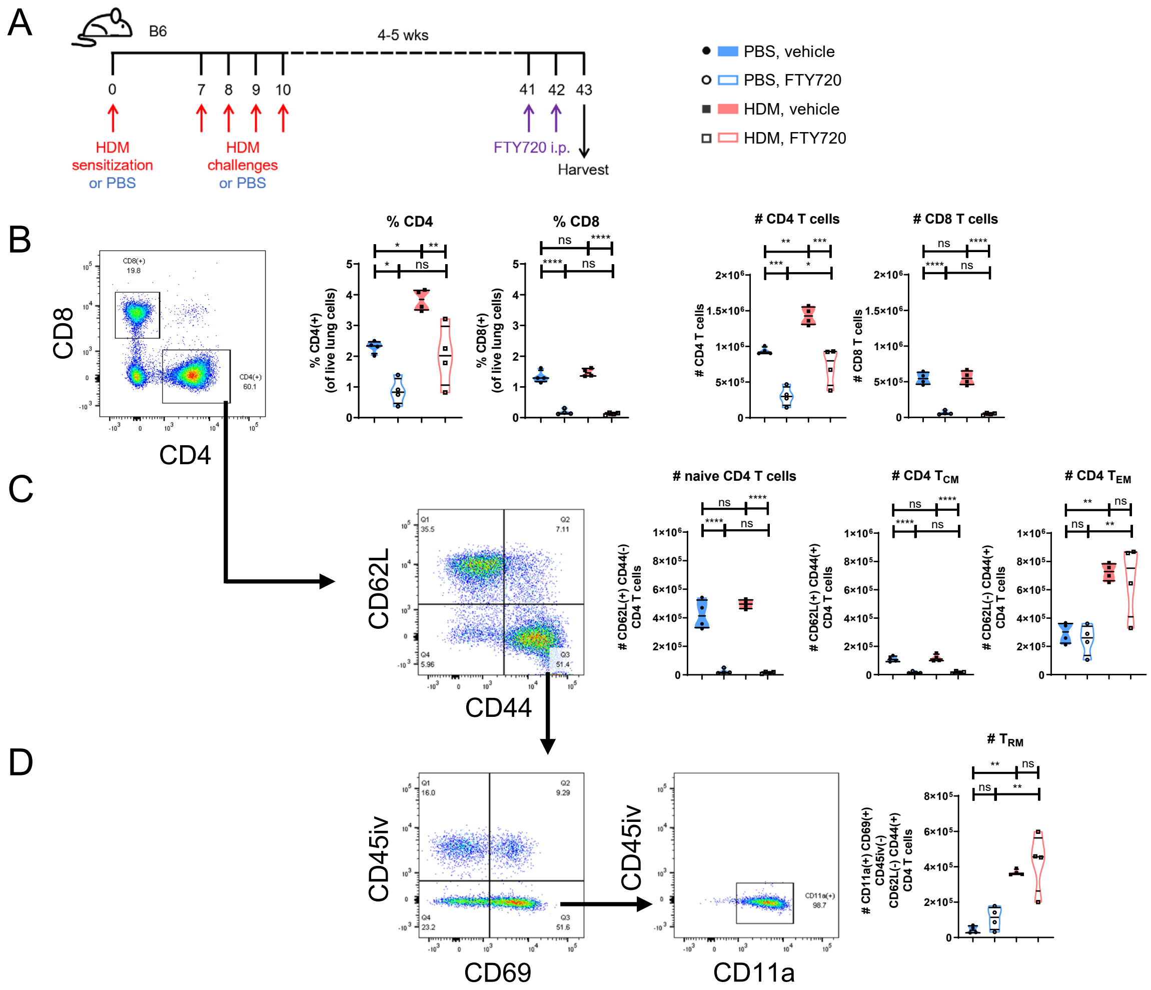

Figure S2, related to Fig. 1: Systemic treatment with FTY720 depletes circulating T cell populations but leaves lung T_{RM} intact. (**A**) Schematic depicting experimental protocol. After sensitization and challenge with HDM and resolution of the acute response, mice underwent treatment with 25 ug FTY720 administered intraperitoneally daily until sacrifice. (**B**) Examination of the lung T cell populations revealed considerable depletion of the CD4 compartment and near-total depletion of the CD8 compartment. (**C**) CD4 T cells were defined as naïve (CD44(low)CD62L(high)), central memory (T_{CM}, CD44(high)CD62L(high)), or effector memory (TEM, CD44(high)CD62L(low)). FTY720 depleted the naïve and T_{CM}, but not T_{EM}. (**D**) CD4 T_{RM} were defined as CD4 T_{EM} which expressed CD69 and CD11a, but did not display intravascular CD45 staining. CD4 T_{RM} were not depleted by FTY720 treatment. Data represent one experiment with n = 4 mice per group, and statistics (ordinary one-way ANOVA with Tukey's multiple comparisons test) were performed in GraphPad Prism. Bar represents the mean ± SEM (*, $p < 0.05$; **, $p < 0.01$; ***, $p <0.001$; **** $p < 0.0001$; ns, not significant).

Figure S3

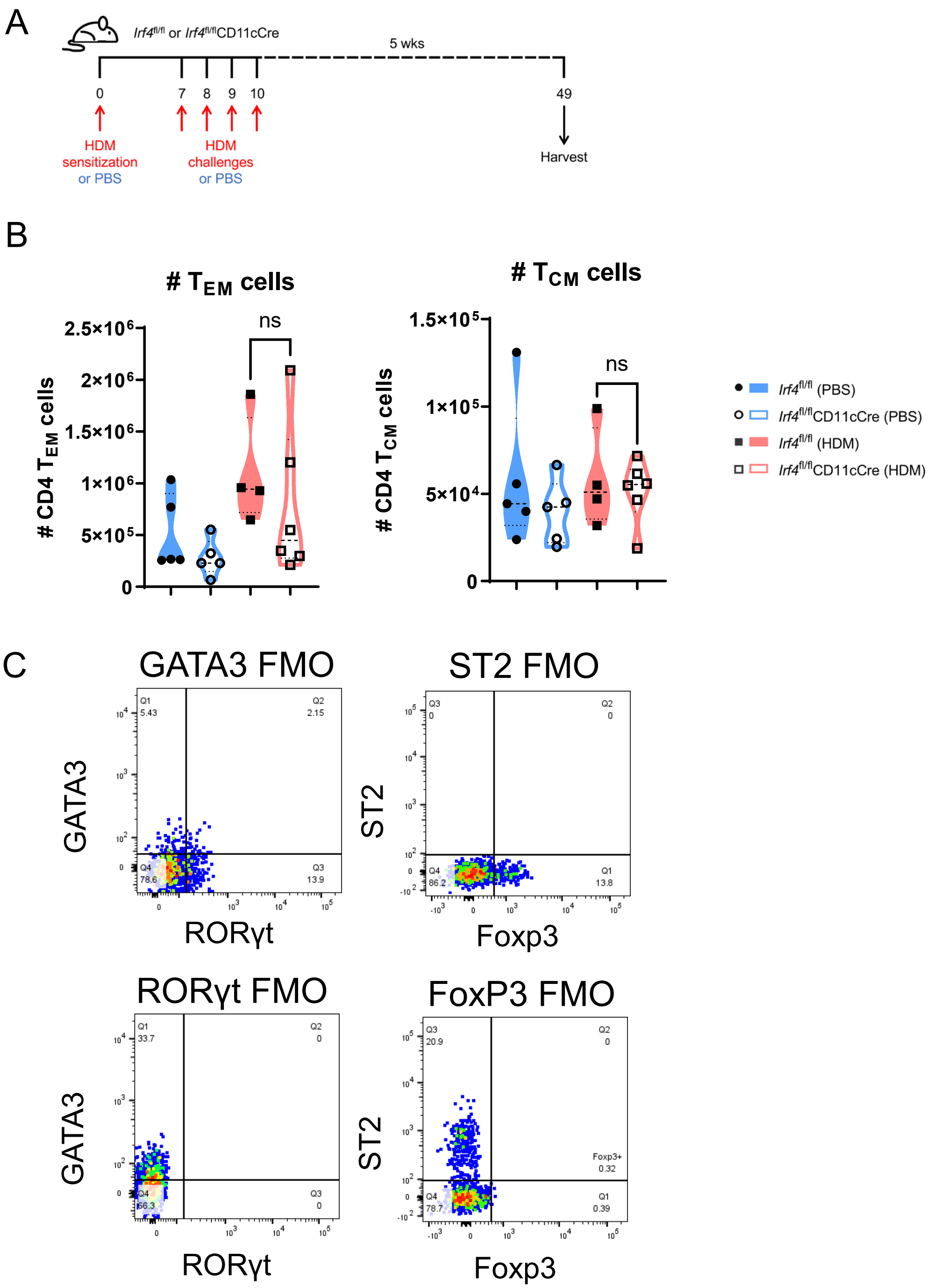

Figure S3, related to Fig. 2: Lungs of mice with IRF4-deficient DCs contain equal numbers of effector memory and central memory T cells during the memory phase. (**A**) Schematic of experimental protocol for resting memory lung analysis (**B)** Quantification of total CD4 effector memory (T_{EM}) and central memory (T_{CM}) cells in the lungs of mice with resting memory to HDM. (**C**) Flow plots demonstrating fluorescence-minus-one controls (FMOs) for the transcription factors GATA3, FoxP3, RORγt, and the IL33 receptor, ST2. Cells were pre-gated on live, CD3+CD4+CD44+CD62L-. Data represent two experiments with n = 4-6 mice per group, and statistics (ordinary one-way ANOVA with Tukey's multiple comparisons test) were performed in GraphPad Prism. Bar represents the mean ± SEM (ns, not significant).

Figure S4

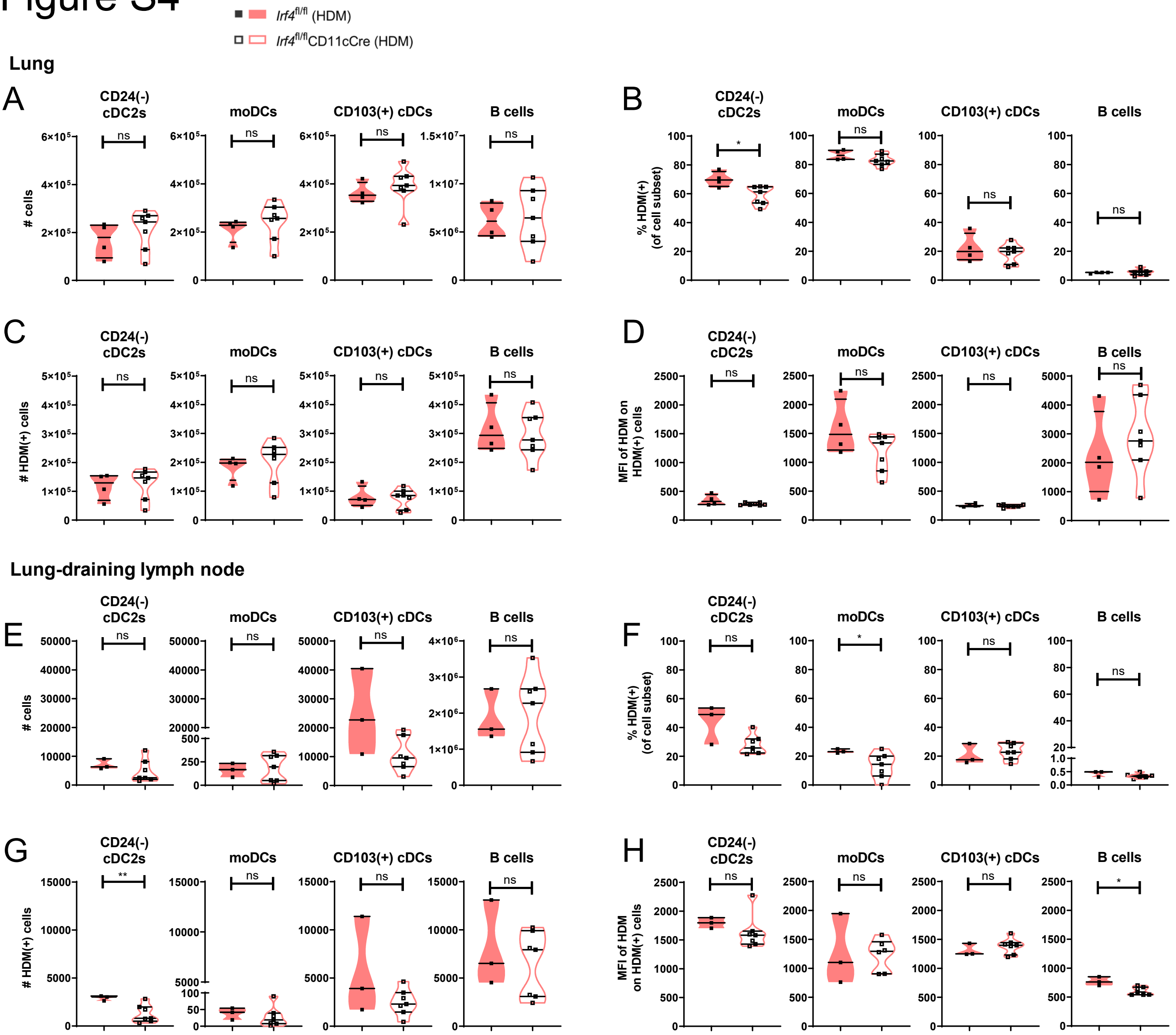

Figure S4, related to Fig. 4: (**A**) Number of lung CD24(-) cDC2s, moDCs, CD103(+) cDCs, and B cells (**B**) HDM uptake by lung CD24(-) cDC2s, moDCs, CD103(+) cDCs, and B cells, (**C**) Number of HDM-bearing lung CD24(-) cDC2s, moDCs, CD103(+) cDCs, and B cells (**D**) MFI of HDM on HDM-bearing lung CD24(-) cDC2s, moDCs, CD103(+) cDCs, and B cells (**E**) Number of LN CD24(-) cDC2s, moDCs, CD103(+) cDCs, and B cells (**F**) HDM uptake by LN CD24(-) cDC2s, moDCs, CD103(+) cDCs, and B cells, (**G**) Number of HDM-bearing LN CD24(-) cDC2s, moDCs, CD103(+) cDCs, and B cells (**H**) MFI of HDM on HDM-bearing LN CD24(-) cDC2s, moDCs, CD103(+) cDCs, and B cells. Data are representative of at least 2 independent experiments with $n \geq 4$ mice per group, and statistics (unpaired t test with Welch's correction) were performed in GraphPad Prism. Bar represents the mean ± SEM (*, $p < 0.05$; **, $p < 0.01$; ns, not significant).

Figure S5

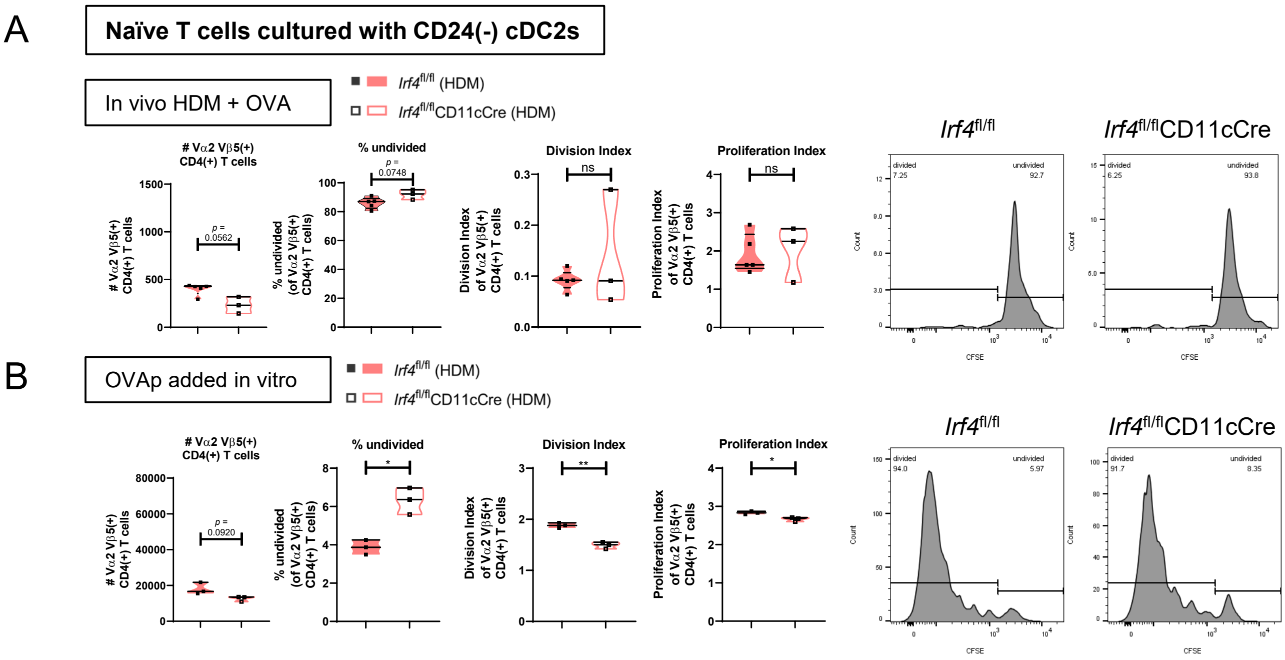

Figure S5, related to Fig. 5: CD24(-) cDC2s are poor stimulators of naïve T cells in response to HDM. CD24(-) cDC2s were sorted from the lungs on day 1 following in vivo sensitization to HDM + OVA, then were cocultured with CFSE-labeled T cells from naïve OTII mice. Depicted are the number of OTII cells after culture, % undivided, division index, proliferation index, and CFSE dilution histograms for (**A**) in vivo HDM + OVA sensitized CD24(-) cDC2s or (**B**) with $OVA_{323\text{-}339}$ peptide added. Data are representative of at least 2 independent experiments with n ≥ 3 wells per group, and statistics (unpaired t test with Welch's correction) were performed in GraphPad Prism. Bar represents the mean ± SEM (*, $p < 0.05$; **, $p < 0.01$; ns, not significant).

Figure S6

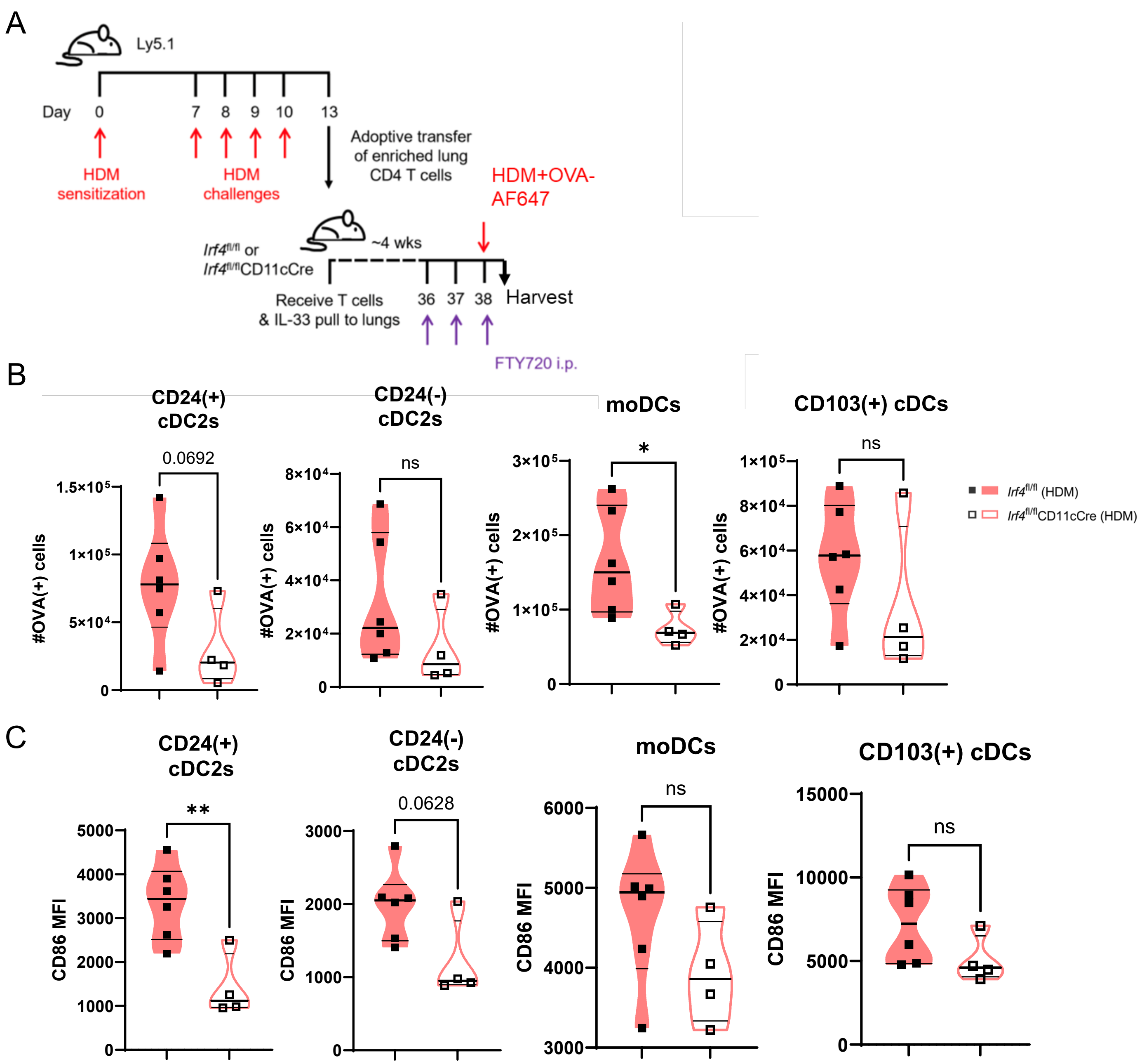

Figure S6, related to Fig. 6: (**A**) Schematic depicting experimental protocol. After sensitization and challenge with HDM, lung CD4 T cells were adoptively transferred into naïve Ly5.1(+) recipients and recruited to the lung via i.t. administration of IL-33. After resting for 4 weeks, mice received three doses of FTY720 before i.t. administration of HDM and OVA-AF647. Mice were sacrificed 18h after HDM+OVA treatment. (**B**) Number of OVA-bearing lung CD24(+) cDC2s, CD24(-) cDC2s, moDCs, and CD103(+) cDCs, (**C**) CD86 expression by lung CD24(+) cDC2s, CD24(-) cDC2s, moDCs, and CD103(+) cDCs. Data are from one experiment with n ≥ 4 mice per group, and statistics (unpaired t test with Welch's correction) were performed in GraphPad Prism. Bar represents the mean ± SEM (*, $p < 0.05$; **, $p < 0.01$; ns, not significant).

Supplementary Table 1

Anti mouse	Clone	Manufacturer(s)
CD3	145-2C11	BD Biosciences, Biolegend
	17A2	Biolegend
CD4	GK1.5	BD Biosciences
	RM4-5	Biolegend
CD8	53-6.7	Biolegend
CD11a	H155-78	Biolegend
CD11b	M1/70	BD Biosciences, Biolegend
CD11c	N418	Biolegend
CD19	6D5	Biolegend
CD24	M1/69	BD Biosciences, Biolegend
CD44	IM7	Biolegend
CD45	30-F11	Biolegend
CD45.1	A20	Biolegend
CD45.2	104	Biolegend
CD62L	MEL-14	eBioscience, Biolegend
CD69	H1.2F3	eBioscience, BD Biosciences, Biolegend
CD80	16-10A1	Biolegend
CD86	GL-1	Biolegend
CD88	20/70	BD Biosciences, Biolegend
CD103	2E7	eBioscience, Biolegend
CCR7	4B12	eBioscience
Foxp3	FJK-16s	eBioscience
GATA3	16E10A23	Biolegend
γ/δ TCR	GL3	Biolegend
IL-4	11B11	eBioscience, Biolegend
IL-5	TRFK5	Biolegend
IL-13	eBio13A	eBioscience
I-A/I-E	M5/114.15.2	Biolegend
RORγt	Q31-378	BD Biosciences
SiglecF	E50-2440	BD Biosciences
ST2	DIH4	Biolegend
Va2 TCR	B20.1	eBioscience, BD Pharmingen
Vβ5.1, 5.2 TCR	MR9-4	eBioscience, BD Pharmingen
PE conjugated streptavidin		eBioscience
Brilliant Violet 711 streptavidin		Biolegend
APC/Cy7 streptavidin		Biolegend

HVTN 405/HPTN 1901 Study Team

The HVTN 405/HPTN 1901 Study Team includes the following: manuscript coauthors, and Katherine Gill, Julia Hutter, John Hural, William O. Hahn, Jen Hanke, Lisa Sanders, Laurie Rinn, Theresa Wagner, Doug Grove, Gail Broder, Ro Yoon, Robert De La Grecca, Carissa Karg, Alison Ayres, Vicky Kim, Megan Jones, Nick Maurice, Meg Trahey, Leigh Fisher, Laura Polakowski, David Montefiori and Simba Takuva
